# Design, synthesis, docking, and antidepressant activity evaluation of isatin derivatives bearing Schiff bases

**DOI:** 10.22038/IJBMS.2023.68363.14916

**Published:** 2023-04

**Authors:** Azadeh Mesripour, Elham Jafari, Mohammad Reza Hajibeiki, Farshid Hassanzadeh

**Affiliations:** 1 Department of Pharmacology and Toxicology, School of Pharmacy and Pharmaceutical Sciences, Isfahan University of Medical Sciences, Isfahan, Iran; 2 Isfahan Pharmaceutical Science Research Center, School of Pharmacy and Pharmaceutical Sciences, Isfahan University of Medical Sciences, Isfahan, Iran; 3 Department of Medicinal Chemistry, School of Pharmacy and Pharmaceutical Sciences, Isfahan University of Medical Sciences, Isfahan, Iran

**Keywords:** Antidepressive agents, Inhibitors, Isatin, Monoamine oxidase, Obsessive-compulsive - disorder Schiff-base

## Abstract

**Objective(s):**

Depression is a prevalent psychiatric disorder. Treatment of depression is still a challenge due to the lack of response of some patients to a variety of available medications and side effects. Isatin is an interesting molecule with diversified biological effects. It also participates in many synthetic reactions, as a precursor molecule. In this study, a new series of N-alkyl and N-benzyl isatin derivatives bearing Schiff bases were synthesized and screened for antidepressant activities in mice.

**Materials and Methods:**

The synthesis was initiated by N-alkylation and N-benzylation of isatin by an alkylation reaction to give N-substituted isatins. 2-(Benzyloxy) benzohydrazide derivatives were synthesized by treating methyl2-hydroxybenzoate with benzyl bromide or 4-chlorobenzyl bromide which was followed by a reaction with hydrazine hydrate to provide acid hydrazide derivatives. The final compounds were obtained by condensation of N-substituted isatins with 2-(benzyloxy) benzohydrazide derivatives as Shiff-base products. Compounds were evaluated for antidepressant activities in mice by the locomotor activity, marble burying test, and forced swimming test. Monoamine oxidase–A (MAO–A) enzyme has been used for molecular docking studies.

**Results:**

Compounds 8b and 8e in both doses, and 8 c in the lower dose, reduced immobility time during the forced swimming test relative to the control group. All preparations reduced the number of marbles buried compared with the control group. The highest docking score was -11.01 kcal/mol for compound 8e.

**Conclusion:**

N-Benzylated-isatin (8b, 8e) and N- acetic acid ethyl ester -isatin derivatives (8c) showed more effective antidepressant activity compared with N-phenyl acetamide isatin derivatives. Docking results relatively confirm the pharmacological results.

## Introduction

World Health Organization (WHO), has stated equal importance for mental and physical health, and these two areas are inseparable by their interaction (1). Mental illness disrupts education and reduces the quality of life therefore; a comprehensive study of the factors that cause them and the use of appropriate decisions to eliminate them is very important (2). Major depressive disorder (MDD) is one of the widespread psychiatric disorders. Risk factors for MDD include conditions associated with chronic inflammation and immunological changes such as rheumatoid arthritis, obesity, and diabetes. Monoamine is the most important hypothesis for this disorder so far (2). A broad range of antidepressant agents were introduced in the market but many patients will not respond to them or show side effects. Therefore, development of agents for the treatment of psychiatric disorders has played a challenging role in medicinal chemistry research (3).

Isatin is a privileged building block with wide pharmacological properties including anti-microbial (4-6), anti-convulsant (7, 8), antidepressant (7,8), anti-inflammatory (9), anti-Parkinson, and anti-Alzheimer (10) effects. Isatin derivatives, such as hydrazones, and Schiff’s and Mannich bases, have shown numerous CNS activities (11). Hydrazine-based compounds have displayed a selective inhibitory activity against monoamine oxidase enzyme B (MAO-B). Isatin acts as a regulator of the biological system in the brain (8, 12). The presence of a significant amount of endogenous isatin in tissues such as the brain is an explanation for the possible role of this substance in central nervous system (CNS) function (13). The proposed mechanisms for the involvement of this substance in CNS action include the possible effect on cannabinoid receptors (14) and inhibition of MAO B (3). The basal tissue concentrations of isatin are in the range of <0.1–1 µM, and in some tissues, the concentration may rise to 10 µM (15). Isatin is a reversible inhibitor of (MAO B), and protection of the active site of the enzyme is associated with its neuroprotective effects. It has been shown that administration of a high dose of isatin protected MAO B against irreversible inactivation of the enzyme by MPTP (1-methyl-4-phenyl-1,2,3,6-tetrahydropyridine) and the induction of locomotor impairments known in Parkinson’s disease (16). Literature surveys have shown that isatin can increase the level of monoamines in CNS, which could be the result of MAO inhibition (4, 7). Isatin substituted at C5 and C6 identified as reversible inhibitors of MAO-(A and B), especially C5- and C6-benzyloxy substituted isatin have been introduced as MAO-B inhibitors (4, 17). Jupally *et al*. synthesized isatin--N -benzalaminothiazol-hydrazone derivatives that showed CNS activities (3). Vishnu *et al*. synthesized isatin-based hydrazones to develop dual inhibitory activity against both MAO and ChE (choline esterase) enzymes (17). According to the important role of isatin derivatives, especially isatin-hydrazone in MAO inhibition, in this study, isatin-Schiff bases derivatives as effective small molecules with promising biological activity were subjected to synthesis and antidepressant activity evaluation. Monoamine oxidase–A (MAO–A) enzyme (PDB ID: 2BXS) has been used for molecular docking studies.

## Materials and Methods

All materials and reagents were purchased from commercial suppliers like the Merck (Germany) company. To analyze reactions, silica gel 60 F_254_ (Germany) was used. Proton nuclear magnetic resonance (HNMR) spectra were recorded using a Bruker 400 MHz spectrometer (Germany). Prepared KBr discs were used for recording Infrared (IR) spectra with a WQF-510 Fourier-transform IR (FT-IR) spectrophotometer (China). Melting points were measured with electrothermal 9200 melting point apparatus (United Kingdom) and are uncorrected. Mass spectra were performed on an Agilent Technologies 5975C mass spectrometer (USA).

***Chloro-N-phenylacetamide (2)***

**Scheme 1:**
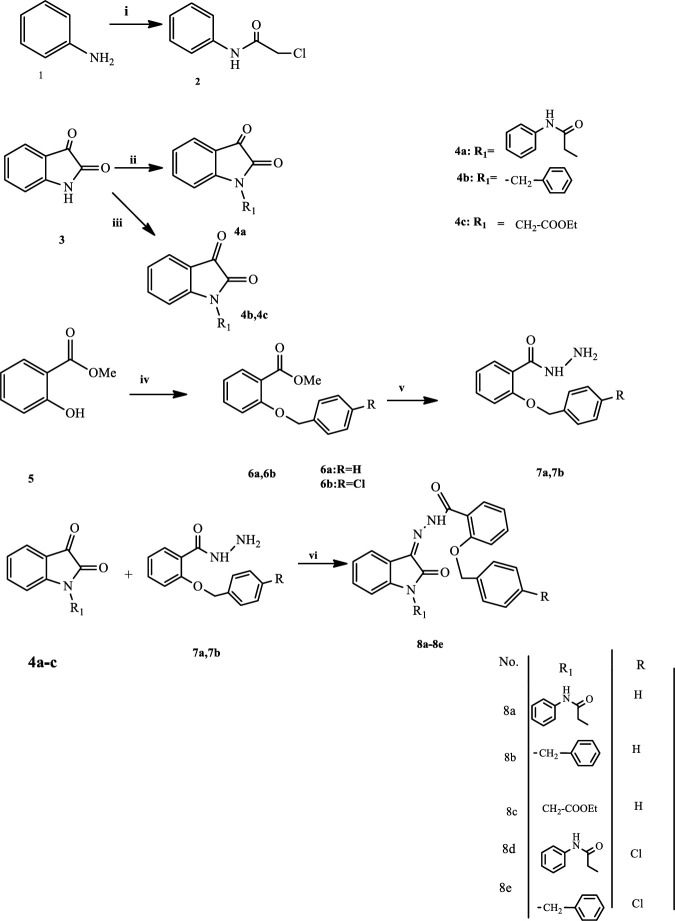
(i): Glacial acetic acid/saturated sodium acetate solution, chloroacetyl chloride, RT, 1 hr (ii): DMF, K_2_CO_3_, 2-chloro-N-phenylacetamide, KI, 80 °C, 3 hr (iii): Acetonitrile, K_2_CO_3_, benzyl bromide or ethyl 2-chloroacetate, reflux, 5 hr (iv): Acetonitrile, K_2_CO_3_, benzyl bromide or 4-chlorobenzylbromide, reflux, 24 hr (v): Absolute ethanol, hydrazine hydrate, reflux, 9 hr (vi): Absolute methanol, glacial acetic acid, reflux, 24 hr

Chloroacetyl chloride (0.02mol) was added to a solution of aniline (0.02 mol) in a mixture of glacial acetic acid/ saturated solution of sodium acetate (20ml/20ml). Stirring at room temperature was used to carry out the reaction. Filtered precipitation was washed with water and crystallized from ethanol to give a white solid (8, 18) (Scheme 1).

***Synthesis of (4a)***

Potassium carbonate (0.02 mol) was added to isatin solution **3** (0.01 mol) in dimethyl formamide (DMF) (30 ml) and the colored suspension was stirred at room temperature for one hour. Compound 2 (0.01 mol) and potassium iodide (0.01 mol) were added, and the reaction mixture was stirred at 80 °C for 3 hr until the reaction was completed. The reaction mixture was spilled into three times its volume of water, and then diluted hydrochloric acid was added and stirred in order to adjust the pH value around three and four. The precipitated product was ﬁltered and washed with cold water, and crystallized from methanol (8, 18-20) (Scheme 1).

***Synthesis of 1-benzylindoline-2, 3-dion (4b)***

Isatin **3** (0.01mol) was stirred and dissolved in acetonitrile (30 ml). In the presence of dry potassium carbonate (0.02mol), benzyl bromide (0.01 mol) was added, and the mixture was refluxed for 5 hr (21, 22). After monitoring the reaction with TLC, the reaction mixture was filtered and the solid crystallized from ethylacetate/petroleum ether (1:1, v/v) to get compound 4b (Scheme 1).

***Synthesis of ethyl 2-(2, 3-dioxoindolin-1-yl) acetate (4c)***

To a solution of isatin 3 (0.01 mol) and anhydrous potassium carbonate (0.02 mol) in acetonitrile (30 ml), ethyl 2-chloroacetate (0.01 mol) was added and the resultant mixture was refluxed for 5 hr (21, 23). Upon completion of the reaction, the mixture was filtered and crystallized by using ethyl acetate/petroleum ether (3:1, v/v) to afford compound 4c (Scheme 1).

***Synthesis of methyl 2-(benzyloxy) benzoate derivatives (6a, 6b)***

A mixture of methyl2-hydroxybenzoate 5 (0.02 mol) and potassium carbonate (0.04 mol) in acetonitrile (15 ml) was stirred at room temperature and benzyl bromide or 4-chlorobenzyl bromide (0.02 mol) was added and heated to 85 °C for 24 hr (22). To the cooled reaction mixture was added water and dichloromethane, and organic phase was separated and evaporated under vacuum and purified with ethyl acetate to give 6a and petroleum ether to give 6b (Scheme 1). 

***Synthesis of 2-(benzyloxy)benzohydrazide derivatives (7a, 7b)***

Methyl 2-(benzyloxy) benzoate derivatives (0.01 mol) were added dropwise to a solution of hydrazine hydrate (0.02 mol) in ethanol (30 ml) (9). The solution was refluxed for 9 hr to complete the reaction. Then the solvent was removed and the obtained precipitation was filtered and puriﬁed by recrystallization in ethyl acetate/petroleum ether (Scheme 1).

***Synthesis of final compounds 8 (a-e)***

An equimolar amount of the appropriate substituted isatin 4 (a-c) (0.01 mol) and compounds 7a or 7b were added to methanol (20 ml) containing glacial acetic acid and refluxed for 24 hr. The mixture was filtered and the solid was recrystallized from methanol to give 8a, 8c, 8d, and n-hexane/methanol to furnish 8b and 8e compounds. 

***Pharmacological studies***

Male NMRI mice (weighing 23–29 g, 6–8 weeks old) were used for the assay. Six mice were kept together in each cage at room temperature 21± 2 ºC on a 12 hr light and 12 hr dark cycle (lights on at 06:00) with free access to standard mice chow and tap water. The behavioral experiments were performed from the morning until 1:00 PM. The animal experiments were performed consistent with the Guidelines for Using Laboratory Animals provided by Iran National Committee for Ethics in Biomedical Research (Ethics code: IR.MUI.RESEARCH.REC.1399.6.27; approval date: 12/28/2020). Animal welfare was considered carefully and efforts were made to reduce the number of animals used in the experiments.

The synthesized compounds after being dispersed in 0.1% tween 80 (Merck, Germany) in normal saline (v/v), were injected (25 and 50 mg/kg) intraperitoneally and individually 45 min before starting the tests. The control group received 0.1% tween 80 in normal saline. Imipramine HCl (Sigma, India) (10 mg/kg) was used as the standard antidepressant drug; the volumes of all injections were 10 ml/kg. First the locomotor activity, then the marble burying test (MBT), and lastly the forced swimming test (FST) were carried out for each animal.

***Locomotor activity test***

The locomotor activity was determined in an automated open field apparatus (40×40×40 cm^3^), (BorjSanat, Iran), surrounded by dark walls and a white floor that was divided into 15 zones by the red beams crossing the floor. Each mouse was placed in the corner of the apparatus and allowed to explore it for 3 min. The total activity count was measured by summing the zone entries (horizontal movements recorded automatically by crossings of the red beams) and the rears on hind legs (vertical movements, recorded manually) (24).

***Marble burying test***

This test was conducted in an open, transparent glass box (29 cm×34 cm, depth 18 cm) covered with 5 cm deep small sawdust chips. Twelve glass marbles (15 mm diameter) were evenly distributed on the sawdust, each animal was placed in the field for 20 min, and the number of marbles buried (MB) was counted to evaluate the obsessive-compulsive behavior in mice (24); animals with less anxiety level dig less marble. 

***Forced swimming test***

The mice were forced to swim for 6 min in a 2-liter Pyrex beaker (diameter 12.5 cm, depth 12 cm) filled with water (25 ºC). The first 2 min was considered for the habituation period and immobility time was measured during the last 4 min. The time the animal had no extra activity except that necessary for keeping the head out of the water was identified as the immobility time. An increase in the immobility time reflects the despair behavior. The entire experiment was recorded by a camera (25). In the end, the animals were dried carefully to avoid hypothermia.

The results were expressed as group mean ± standard error of the mean (SEM). The results of synthesized compounds were compared with the control group by one-way analysis of variance (ANOVA) followed by Tukey’s multiple comparison tests and *P*-values less than 0.05 were defined as statistically significant. The software programs used for data analysis and making graphs were Excel 2010 and GraphPad Prism 8.

***Docking study***

3D structure of the monoamine oxidase-A enzyme has been obtained from the Protein Data Bank (PDB ID: 2BXS) with resolution 3.15 Å. The PDBQT file of the enzyme was prepared according to previous studies (2). Three-dimensional structures of the ligands were drawn in HyperChem 7.0 software, then the ligands were optimized using a molecular mechanical force field. The ultimate conformations were calculated by AM1 as a semi-empirical method, and molecular structures were optimized using the Polak Ribiere conjugate gradient algorithm. Optimized structures were used as inputs for Auto Dock tools to prepare PDBQT files of ligands. Grid box dimensions were 60×60×60 with a 0.375Å grid points spacing. The binding location of the reference ligand was selected as a binding site for finding the best pose of all ligands. The molecular docking technique was conducted using the Autodock4.2 software package, with the implemented empirical free energy function and the Lamarckian genetic algorithm (2, 26). 

## Results

**2-(Benzyloxy) benzohydrazide (7a)**

Yield: 67.5%, white solid, m.p. 72-74 °C; IR (cm^-1^): 3330, 3238 (NH), 2939 (C-H, aliphatic), 1624 (C=O), 1215, 1293 (C-O).^1^HNMR (400 MHz; DMSO- d_6_) δ ppm : 9.25(1H, s, NH),7.67 (1H, d, *J*=8Hz, H-Ar),7.50 (2H, d, *J*=8Hz, H-Ar), 7.45-7.33 (4H, m, H-Ar), 7.18 (1H, d, *J*=8Hz, H-Ar), 7.03 (1H, t, *J*=8Hz, H-Ar), 5.25 (2H, s, CH_2_), 4.53 (2H, s, NH_2_).

***2-(Benzyloxy)-N’-(2-oxo-2-(phenylamino) ethyl) indolin-3-ylidene) benzohydrazide (8a)***

Yield: 66%, yellow solid, m.p. 236 °C decomposed; IR (cm^-1^): 3509, 3299 (NH), 3100 (C-H, aromatic), 2918 (C-H, aliphatic), 1715 (C=O), 1680 (C=N), 1607, 1481 (C=C), 1289 (C-O); ^1^HNMR (400 MHz; DMSO- d_6_) δ ppm : 11.75 (1H, s, NH), 10.37 (1H, s, NH), 7.59-7.57 (3H, m, *J*=8Hz, H-Ar), 7.41-7.37(3H, m, H-Ar), 7.34-7.30 (3H, m, H-Ar), 7.27-7.15 (6H, m, H-Ar), 7.09-7.06 (3H, m, H-Ar), 5.43 (2H, s, CH_2_), 4.63 (2H, s, CH_2_).MS (m/z): 504(M^+^); calculated 504.54 g/mol.

***N’-(1-Benzyl-2-oxoindolin-3-ylidene)-2-(benzyloxy)benzohydazide (8b)***

Yield: 86%, yellow solid, m.p. 186-188 °C; IR (cm^-1^): 3416 (NH), 3108 (C-H, aromatic), 2907(C-H, aliphatic), 1735, 1674 (C=O), 1601, 1477 (C=C), 1289 (C-O).^ 1^HNMR (400 MHz; DMSO- d_6_) δ ppm : 14.38 (1H, s, NH), 8.09 (1H, d, *J*=8Hz, H-Ar), 7.71-7.67(2H, m, H-Ar), 7.56-7.52 (2H, m, H-Ar), 7.41-7.26 (10H, m, H-Ar), 7.19-7.1 (2H, m, H-Ar), 7.07 (1H, d, *J*=8Hz, H-Ar), 5.62(2H, s, CH_2_), 4.99 (2H, s, CH_2_). MS (m/z): 461(M^+^); calculated 461.51 g/mol.

***3-[(2-Benzyloxy-benzoyl-hydrazono]-2-oxo-2, 3-dihydro-indol-1-yl]-acetic acid ethyl ester) (8C)***

Yield: 53%, yellow solid, m.p. 145-147 °C; IR (cm^-1^): 3501, 3201(NH), 2934 (C-H, aliphatic), 1709, 1759, 1677 (C=O), 1604(C=C), 1286, 1231(C-O).^ 1^HNMR (400 MHz; DMSO-d_6_)δ ppm : 14.35 (1H, s, NH), 8.01 (1H, d, *J*=8Hz, H-Ar), 7.70-7.69 (2H, m, H-Ar), 7.56-7.45 (3H, m, H-Ar), 7.33 (2H, t, *J*=8Hz, H-Ar), 7.29-7.24 (3H, m, H-Ar), 7.19 (1H, d, *J*=8Hz, H-Ar), 7.12 (1H, t, *J*=8Hz, H-Ar), 5.59 (2H, s, CH_2_), 4.69 (2H, s, CH_2_), 4.17 (2H, q, *J*=8Hz, CH_2_), 1.19 (3H, t, *J*=8Hz, CH_3_). MS (m/z): 457 (M^+^); calculated 457.48 g/mol.

***2-(4-Chlorobenzyloxy)-N’-(2-oxo-1-(2-oxo-2-(phenylamino)ethyl)indolin-3-ylidene)benzohydrazide (8d)***

Yield: 68%, yellow solid, m.p. 240 °C decomposed; IR (ν_max_ cm^-1^): 3436, 3261 (NH), 2934 (C-H, aliphatic), 1672 (C=O), 1607 (C=C), 1469 (C-H, aliphatic), 1205 (C-O), 791 (C-Cl).^ 1^HNMR (400 MHz; DMSO- d_6_) δ ppm : 14.27 (1H, s, NH), 10.34 (1H, s, NH), 8.08 (1H, d, *J*=8Hz, H-Ar), 7.70 (1H, d, *J*=8Hz, H-Ar), 7.57-7.46 (5H, m, H-Ar), 7.37-7.29 (4H, m, H-Ar), 7.23-7.07 (6H, m, H-Ar), 5.56 (2H, s, CH_2_), 4.67 (2H, s, CH_2_), MS (m/z): 538 (M^+^), 540 (M+2); calculated538.9 g/mol. 

***2-(4-Chlorobenzyloxy)-N’-(1-benzyl-2-oxoindolin-3-ylidene) benzohydrazide (8e)***

Yield: 72%, yellow solid, m.p. 188-190 °C; IR (ν_max_ cm^-1^):3474, 3218 (NH), 3023 (C-H, aromatic), 2835 (C-H, aliphatic), 1681(C=O), 1605(C=C), 1229(C-O).^ 1^HNMR (400 MHz; DMSO- d_6_) δ ppm :14.38 (1H, s, NH), 8.1 (1H, d, *J*=8HZ, H-Ar), 7.73-7.67 (2H, m, H-Ar), 7.57-7.55 (2H, m, H-Ar),7.42-7.36 (3H, m, H-Ar) ,7.35-7.31 (3H,m , H-Ar), 7.30-7.26 (3H, m, H-Ar), 7.19-7.12 (2H, m, H-Ar), 7.07(1H,d,* J*=8Hz, H-Ar), 5.6 (2H, s, CH_2_),4.98 (2H, s, CH_2_), MS (m/z): 495 (M^+^), 497 (M+2), calculated 495 g/mol. 

***Pharmacological results***

**Figure 1:**
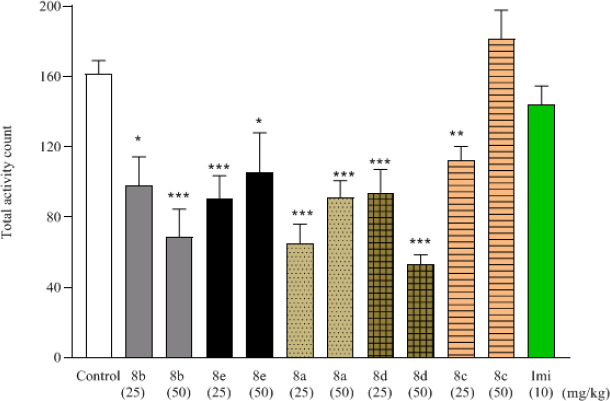
Effect of different treatments of synthesized compounds (8a-8e) (25 mg/Kg and 50 mg/Kg) and imipramine (10 mg/Kg) on locomotor activity. Group mean±SEM (n=6). *= *P*<0.05, **=*P* <0.01, and ***= *P*<0.001 compared with the control group (10 ml/kg). Imi= imipramine

As shown in Figure 1 all compounds except **8c** (50 mg/kg) significantly reduced total activity count compared with the control group (161.5±7.5 count). **8d** (50 mg/kg) showed the least locomotor activity (53±5.5 count, *P*<0.001 vs the control group). The locomotor activity of the imipramine group was not different from the control group.

**Figure 2:**
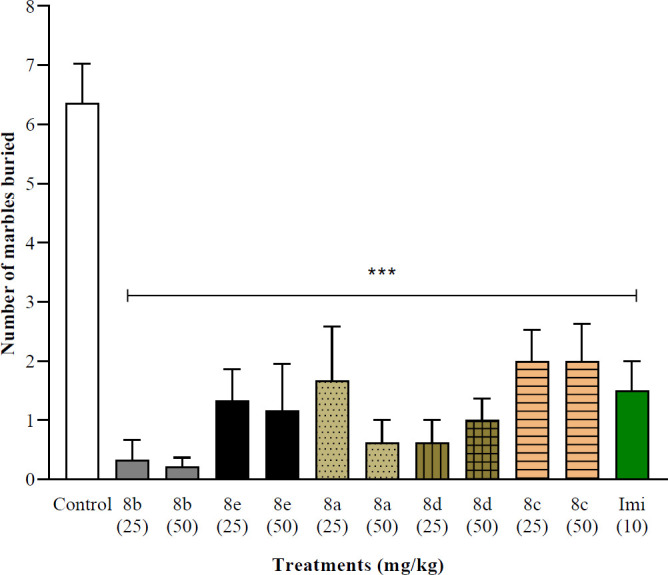
Effect of synthesized compounds (8a-8e) on MBT. Group mean±SEM (n=6). ***= *P*<0.001 compared with the control group (10 ml/kg). Imi= imipramine

Figure 2 shows the MBT results, all the products significantly reduce the number of MB compared with the control group (*P*<0.001, 6.3±0.6 count), these results were similar to the imipramine group.

**Figure 3:**
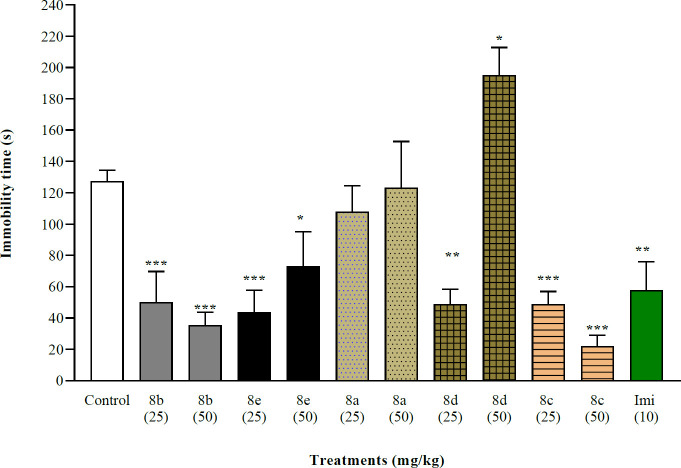
Effect of synthesized compounds (8a-8e) (25 mg/Kg and 50 mg/Kg) and imipramine (10 mg/Kg) on immobility time during the FST. Group mean±SEM (n=6). *=*P*<0.05, **=*P*<0.01, and ***= *P*<0.001 compared with the control group (10 ml/kg). Imi= imipramine

As depicted in Figure 3, the immobility time during FST for both doses (25 and 50 mg/kg) of **8b**, **8e**, and **8c** reduced by about 72–83 %, which was significantly lower than the control group (127.3±7.1 sec) like imipramine. **8d** (25 mg/kg) also significantly reduced the immobility time (48.8±9.5 sec vs control, *P*<0.01), but **8d** (50 mg/kg) significantly increased the immobility time (195.1±2.5 sec vs control, *P*<0.05). While the immobility time of **8a** was not different from the control group.

***Docking results***

**Figure 4.**
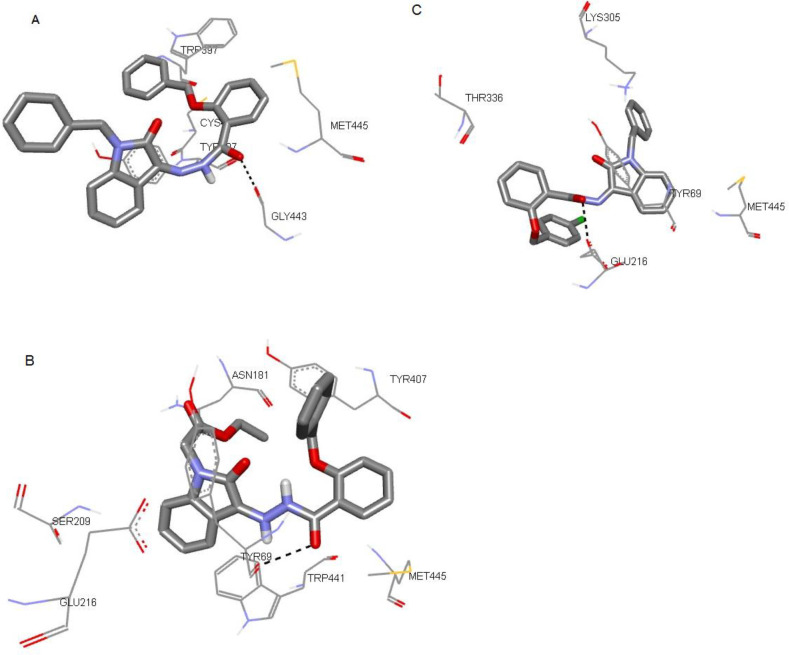
Docked conformation of compounds (A) 8b, (B) 8c, and (C) 8e in the binding site of Monoamine oxidase–A (MAO–A) enzyme

The docking results of compounds including the estimated free binding energy values (kcal/mol), and the interactions with key amino acid residues at the active site of enzymes are expressed in Table 1 and Figure 4. All compounds except **8a** showed hydrogen interactions at the active site of enzymes.

## Discussion

The final compounds **(8a-8e)** were synthesized according to the sequence shown in Scheme 1.

N-alkylation and N-benzylation of isatin were carried out via reaction with 2-chloro-N-phenylacetamide, benzyl bromide, or ethyl 2-chloroacetate in various solvents (DMF or acetonitrile) in the presence of potassium carbonate. The reaction of methyl2-hydroxybenzoate with appropriate benzyl bromide and potassium carbonate in acetonitrile afforded corresponding products **6** (**a, b**), followed by a reaction with hydrazine hydrate to provide acid hydrazide derivatives **7 **(**a,b**). Final derivatives (**8a-8e**) were obtained by condensing N-substituted isatins with 2-(benzyloxy) benzohydrazide derivatives in methanol. The IR spectrum of final compounds (**8a-8e**) showed prominent absorption bond corresponding to N–H at 3509-3201 cm^–1 ^range and carbonyl at 1759-1672 cm^-1 ^range. The presence of aliphatic groups was confirmed by the peak at the 2934-2835 cm^-1 ^range. Ether bands were observed in the 1205-1289 range. Methylene protons were observed as a singlet in ^1^ H NMR spectra around δ 5.62–5.43 ppm and 4.99–4.63 ppm, which corroborated the formation of O-CH_2_ and N-CH_2_, respectively. The final compounds were subjected to evaluation in locomotor activity, MBT, and FST (Figures 1-3).

The locomotor activity assay is performed in many psychological animal studies in order to validate the behaviors observed during the FST or MBT (27), aberrations in locomotor activity might nonspecifically affect the results in the behavioral tests (27). The average number of total activity after individual treatment with all compounds, except **8c** (50 mg/kg) and imipramine, showed a significant decrease compared with the control group (Figure 1). This decrease in the number of activities in the locomotor test indicates the sedative effect of the compounds.

The results shown in Figure 2 demonstrated that all synthesized compounds had a significant difference in MB behavior compared with the control group. The average total number of hidden marbles decreased to less than two marbles after twenty minutes of synthesized compound administration. According to MBT, it seems that these compounds have anti-obsessive compulsive and anti-anxiety effects. The MB behavior appears to be a natural digging behavior in rodents (24), and it has been used as a model for anxiety disorders including obsessive-compulsive disorder (24, 25, 28). Different experiments have shown that antidepressant drugs such as fluvoxamine, venlafaxine, and doxepin decline this behavior (28). 

FST is used globally for high-quantity screening for the antidepressant effect of substances, and natural products in drug discovery programs (29,30). By placing the mouse in an inescapable situation, the water tank, it gradually loses hope to escape, therefore the immobility time presents a measurable endophenotype of depression that is despair behavior (30). The immobility time of animals during FST was reduced significantly following the administration of doses **8b**, **8e,** and **8c** compared with control groups (Figure 3), which presents antidepressant-like effects. Compound **8d** showed antidepressant-like effects both doses. While all these derivatives except a higher dose of **8c** reduced the locomotor activity but this did not interfere with their antidepressant-like effect during FST. Compound **8a** did not show antidepressant effects in this study. 

5-Methyl-2, 3-dioxoindoline-1-yl acetamide derivatives indicated remarkable antidepressant effects in a similar study performed by Zhen *et al.* (8). After using 50 mg/kg of compound **8d** the locomotor activity declined, which could have influenced behavior during FST, thus the immobility time during FST increased up to 53%. Therefore it can be concluded that the induction of sedative effects by **8d** may be due to the presence of chlorine substitution and higher dose injection that may have affected the immobility time during FST. Compounds **8b** and **8e** in both doses, and compounds **8c** and **8d** only in the lower dose, decrease the immobility time of animals during FST and the number of total activity in locomotor tests, which can introduce them as possible antidepressants and sedative agents. While **8c** higher dose showed an antidepressant-like effect without affecting the locomotor activity. Structure-activity relationship observation in these series of compounds revealed that N-benzylated-isatin (**8b**, **8e**) and N-acetic acid ethyl ester-isatin derivatives (**8c**) had a favorable influence on the activity compared with N-phenyl acetamide isatin derivatives ( **8a**, **8d**). The docking results showed that the highest dock score and the lowest Ki (inhibition constant) belong to compounds **8b,** **8d,** and **8e** which Gly 443, Tyr 407, and Glu 216, respectively, have been detected for the formation of hydrogen bonds with the carbonyl group of the Schiff-base moiety. This can relatively confirm the pharmacological results.

**Table 1:** Energy-based interactions and hydrogen bonds for the final compounds Schiffbase derivatives of isatin

Compounds	G Bind(Kcal/mol)Δ	Hydrogen bond (Distance, Å)	Ki
8a	-8.66	-	450.6 (nM)
8b	-9.58	Gly 443	95.7 (nM)
8c	-7.69	Tyr 69	2.2 (µM)
8d 8e	-9.85 -11.01	Tyr407 Glu 216	60.08(nM) 8.5 (nM)

## Conclusion

Isatin scaffold pharmacological significance furnishes enormous opportunities to discover novel compounds with different modes of action. Some of the N-benzylated/N-alkylated isatin derivatives bearing Schiff bases were synthesized and evaluated for antidepressant activity in FST and MBT models. Results showed that while all compounds had possible anxiolytic effects by reducing MB behavior; compounds **8b** and** 8e **in both doses, and **8c** and **8d **only in 25 mg/kg dose had antidepressant-like activity. Based on the obtained results of docking and cytotoxic tests, compound **8e** seems to be a good lead molecule. 

## Authors’ Contributions

EJ contributed to the conception and design of the work, conducting the study, analyzing the data, and drafting and revising the manuscript. FH contributed to the conception of the work, analyzing the data, and revising the draft. MH performed the experiments and analyzed the data. AM contributed to the conception of the work, conducting the study, and revising the draft. All authors agreed with all aspects of the work.

## Conflicts of Interest

None of the authors have any conflicts of interest.
